# Tc17 Cells in Patients with Uterine Cervical Cancer

**DOI:** 10.1371/journal.pone.0086812

**Published:** 2014-02-11

**Authors:** Yan Zhang, Fei Hou, Xin Liu, Daoxin Ma, Youzhong Zhang, Beihua Kong, Baoxia Cui

**Affiliations:** 1 Department of Obstetrics and Gynecology, Qilu Hospital, Shandong University, Jinan, Shandong, People's Republic of China; 2 Department of Obstetrics and Gynecology, People's Hospital, Weifang City, Shandong, People's Republic of China; 3 Jinan Maternity and Children's Hospital, Jinan, Shandong, People's Republic of China; 4 Haematology Oncology Centre, Qilu Hospital, Shandong University, Jinan, Shandong, People's Republic of China; 5 Gynecology Oncology Key Library of Shandong Province, Qilu Hospital, Shandong University, Jinan, Shandong, People's Republic of China; Shanghai Jiao Tong University School of Medicine, China

## Abstract

**Background:**

The existence of Tc17 cells was recently shown in several types of infectious and autoimmune diseases, but their distribution and functions in uterine cervical cancer (UCC) have not been fully elucidated.

**Methods:**

The frequency of Tc17 cells in peripheral blood samples obtained from UCC patients, cervical intraepithelial neoplasia (CIN) patients and healthy controls was determined by flow cytometry. Besides, the prevalence of Tc17 cells and their relationships to Th17 cells and Foxp3-expressing T cells as well as microvessels in tissue samples of the patients were assessed by immunohistochemistry staining.

**Results:**

Compared to controls, patients with UCC or CIN had a higher proportion of Tc17 cells in both peripheral blood and cervical tissues, but the level of Tc17 cells in UCC tissues was significantly higher than that in CIN tissues. Besides, the increased level of Tc17 in UCC patients was associated with the status of pelvic lymph node metastases and increased microvessel density. Finally, significant correlations of infiltration between Tc17 cells and Th17 cells or Foxp3-expressing T cells were observed in UCC and CIN tissues.

**Conclusions:**

This study indicates that Tc17 cell infiltration in cervical cancers is associated with cancer progression accompanied by increased infiltrations of Th17 cells and regulatory T cells as well as promoted tumor vasculogenesis.

## Introduction

Uterine cervical cancer (UCC), the second most prevalent malignancy in women worldwide [1], is considered to be an important immunogenic tumor, as human papillomavirus (HPV) high-risk subtypes cause multistep carcinogenesis from cervical intraepithelial neoplasia (CIN) through carcinoma in situ to invasive cancer and metastatic cancer. Meanwhile, the responses of the host immune systems, especially the cellular immune response, play an important role in the control of both HPV infections and HPV-associated neoplastic formation [2], [3]. Although the cellular adaptive immunity is an important component in the tumor immune surveillance, the mechanisms underlying tumor immunity is not fully understood [4].

The primary cells responsible for the cellular immune response are a set of T subsets, including helper T cells (Th), cytotoxic T cells (Tc), and suppressor T cells (Ts). A recently described Th subset, CD4^+^ T cells with IL-17 production (Th17 cells), has been shown to play an important role in the conditions of inflammation, autoimmunity and allergic reactions [5]–[7]. In a recent study, we observed that Th17 cells were highly enriched in peripheral blood of human UCC patients, and their levels were positively correlated with the status of lymph node metastases and vasoinvasion [8]. However, the subsets of IL-17^+^CD8^+^ T cells (Tc17 cells), recently found in several conditions of infection and autoimmune diseases [9]–[12], have not been fully studied and their biological functions are still lacking.

CD8^+^ cytotoxic T cells (Tc cells) play a crucial role in the host immune response to intracellular pathogens and cancer. Due to the redundant expression of T-box transcription factor Eomes and T-bet, Tc cells are fated to develop into cytolytic effector cells that produce IFN-γ and express granzyme B and perforin to kill the target cells [13], [14]. However, studies of the effects of Tc17 cells on immune responses are scarce. In contrast to classic CD8^+^ Tc cells, Tc17 cells are negative for granzyme B as well as perforin and lacking cytolytic activity *in vitro* [9], [12], [15], and the absence of Eomes in tumor infiltrating lymphocytes is correlated with enhanced lymph node metastasis in colorectal cancers [16]. Nevertheless, it is interesting that after adoptive transfer of highly purified, Ag-specific, IL-17-secreting CD8^+^ T cells to Ag-expressing hosts, the transferred Tc17 cells could convert to IFN-γ-producing effector cells lacking IL-17 expression, which could be accumulated remarkably and cause significant pulmonary pathologic alterations [12] or the regression of large established tumors [15], [17]. These findings may be explained by the existence of the plasticity of Tc17 cells. However, the exact effects of naturally existed Tc17 cells *in vivo* such as in the lung, in the digestive mucosa [18], and in the tumor-bearing mice [19] are still largely unknown. Tc17 cells were recently detected in human hepatocellular carcinoma [20], but data concerning their biological function as well as regulatory mechanisms are still lacking. Here, we aimed to investigate the levels as well as the possible biologic functions of Tc17 cells in UCC, which is known to be a type of highly immunogenic cancer initiated by the persistent infection of high-risk HPV.

In this study, we sought to determine the distribution of Tc17 cells in bothperipheral blood and cervical tissues from healthy controls, CIN and UCC patients. Moreover, to determine the potential roles of Tc17 cells, relationship between Tc17 cells and clinical features of UCC as well as microvessel density in cervical tissues were investigated. Furthermore, combined with our previously report [8], [25], the correlations of the levels of Tc17 cells with Th17 cells or Foxp3-expressing T cells were also determined.

## Design and Methods

### Ethics statement

Enrollment took place between May 2009 and April 2012 in Qilu Hospital, Shandong University. Our research was approved by the Medical Ethical Committee of Qilu Hospital, Shandong University. A written informed consent document was obtained from each participant. Informed consent declared that remnants of the patient's peripheral blood and cervical tissues excised during surgery would be used for the examination of T subsets. These samples were used to identify Tc17 cells in UCC and CIN, and peripheral blood was also drawn from healthy volunteers for this research.

### Patient and control specimens

Tumor or peripheral blood samples were obtained from 88 untreated UCC patients (age range 34–70 years, median 44 years) and 53 untreated CIN patients (28–60 years, median 42 years) with pathologically confirmed active disease at Qilu Hospital, Shandong University. Samples from 46 UCC patients who underwent curative resection, and 28 CIN patients who underwent cervical conization were used for immunohistochemistry. Blood samples from 49 UCC patients and 25 CIN patients were used for flow cytometric analyses. Individuals with concurrent autoimmune disease, active or chronic infection, cardiovascular diseases, connective tissue diseases or a history of malignant tumors were excluded. None of the patients previously received immunosuppressive, radiotherapy or chemotherapy. The characteristics of the studied population are presented in Tables 1 and 2. Blood specimens (n = 28; 26–67 years, median 42 years) and histological specimens of cervices (n = 18; 31–60 years, median 46 years) from healthy women who underwent hysterectomies for benign uterine diseases with no cervical abnormalities were collected. These histological specimens were obtained as Paraffin-embedded blocks from the Department of Pathology of Qilu Hospital, Shandong University.

**Table 1:** Clinical Characteristics of UCC Patients.

Characteristic	Category	*N* = 88 (%)
FIGO stage	I_A_	14 (16)
	I_B_	46 (52)
	II_A_	20 (23)
	II_B_	8 (9)
Histology type	SCC	77 (88)
	ADC/ADSC	11 (12)
Tumor differentiation	Well	14 (16)
	Moderate	33 (37)
	Poor	41 (47)
Lymph node metastases	Positive	26 (30)
	Negative	62 (70)
Tumor size (cm)	<4	48 (54)
	≥4	40 (46)
Infiltration depth (mm)	<15	39 (44)
	≥15	49 (56)
Vasoinvasion	Yes	28 (32)
	No	51 (58)
	Unknown	9 (10)

Abbreviation: FIGO, International Federation of Gynecologists and Obstetricians; SCC, squamous cell carcinoma; ADC, adenocarcinoma; ADSC, adenosquamous carcinoma.

**Table 2:** Clinical Characteristics of CIN Patients.

Category	Subcategory	Result
No. patients		53
Age (y)	Median	42
	Range	28–60
Clinical stage	CINII	23
	CINIII	30

### Flow cytometric analysis of Tc17 cells

To analyze the prevalence of Tc17 cells, IL-17-producing CD8(+) lymphocytes were evaluated by flow cytometry. In brief, heparinized peripheral whole blood (200 µl) with an equal volume of RPMI-1640 medium was incubated for 4 h at 37°C in 5% CO_2_ in the presence of 25 ng/ml of phorbol myristate acetate (PMA), 1 µg/ml of ionomycin, and 1.7 µg/ml monensin (all from Alexis Biochemicals, San Diego, CA). Then, the cells were incubated with PE-Cy5-conjugated anti-human CD3 and FITC-conjugated anti-human CD8 monoclonal antibodies (eBioscience, San Diego, CA) at room temperature in the dark for 15 min to stain the surface. After fixation and permeabilization, according to the manufacturer's instructions, the cells were stained with a PE-conjugated anti-IL-17 monoclonal antibody for 15 min. Isotype controls were used to enable correct compensation and confirm antibody specificity. Stained cells were analyzed by flow cytometric analysis using a FACScan cytometer equipped with CellQuest software (BD Bioscience PharMingen).

### Immunohistochemistry detection of Tc17 cells and microvessel density (MVD)

Paraffin-embedded tissue sections were deparaffinized and rehydrated, followed by microwave antigen retrieval in EDTA (pH 9.1). For immunohistochemical double staining of Tc17 cells, the sections were processed with the Doublestain system (kit-9999), and the following antibodies were used:rabbit anti-human IL-17 antibody (1∶700 v/v; Abcam, USA) and mouse anti-human CD8 antibody (1∶30 v/v; Abcam, USA). For immunohistochemical analysis of microvessel density, the sections were blocked with blocking solution (serum albumin) diluted in phosphate buffer saline (PBS) 3 times for 5 min. Then, the sections were incubated with primary antibody in a moist chamber at 4°C overnight. The CD34 monoclonal antibody (Abcam, USA) was used in this study. After washing 3 times with PBS for 5 min, a horseradish peroxidase (HRP) polymer-linked secondary antibody was added and incubated for 15 min at 37°C. The sections were then visualized with diaminobenzadine (DAB) and counterstained with hematoxylin. Negative control staining was performed with an isotype control and PBS instead of the primary antibody.

### Statistical analysis

Values were expressed as the mean ± SD. The data were assessed by an ANOVA. Comparisons between two groups were assessed by student's *t* test. Correlations between variables were determined using linear regression analysis. All tests were performed using the SPSS 13.0 system. *P*<0.05 was considered statistically significant.

## Results

### Elevated circulating Tc17 cells in untreated UCC and CIN patients

**Figure 1:**
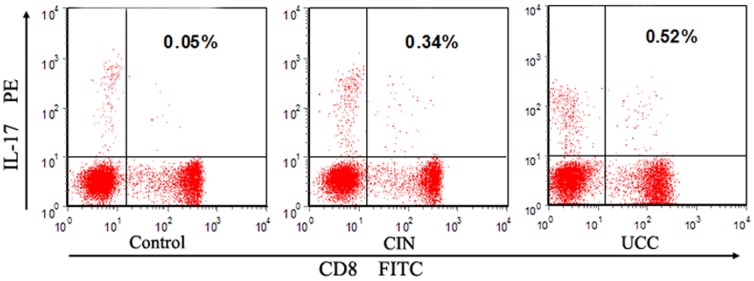
The levels of circulation Tc17 cells in representative controls, CIN patients and UCC patients. Upper right quadrants are the domains of Tc17 (CD8^+^ IL-17^+^) cells and the percentages of them were shown in each panel.

**Figure 2:**
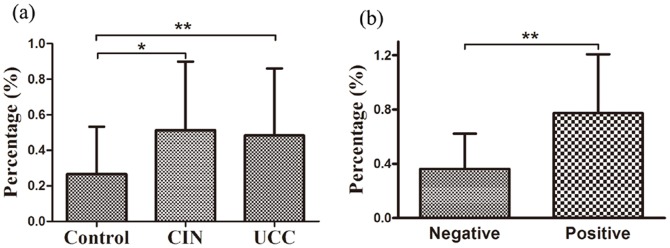
The frequency of Tc17 cells by flow cytometry. (a) Tc17 frequencies in the three groups. Significantly increased Tc17 cells were found in untreated UCC patients (*P* = 0.0076, n = 49) and CIN patients (*P* = 0.0149, n = 25) compared to healthy controls (n = 28). (b) Tc17 frequency in positive or negative lymph node metastases in UCC patients. Compared with patients without lymph node metastases (n = 15), significantly increased Tc17 frequency (*P* = 0.0026) was found in lymph node metastases patients (n = 35). Student's t test was used and bars represent SD, *P<0.05, **P<0.01.

We analyzed the expression of IL-17 on CD8^+^ T cells (Tc17 cells) in peripheral blood using flow cytometry. The expression of typical Tc17 cells in representative patients and controls was shown in Fig. 1. Besides, the percentage of Tc17 cells was significantly higher in UCC patients (0.49±0.37%, *P* = 0.0076) and CIN patients (0.51±0.39%, *P* = 0.0149), compared to that in healthy controls (0.27±0.27%) (Fig. 2a). No significant difference was found between the level of Tc17 cells in CIN and that in UCC patients (*P*>0.05). To investigate the possible role of the increased Tc17 cells, we next sought to determine whether this Tc17 skewing was associated with clinical features of the disease. Notably, we observed a significant increase in the frequency of Tc17 cells in UCC patients with lymph node metastasis (0.77±0.43%; *P* = 0.0026), when compared to that in patients without lymph node metastases (0.36±0.26%) (Fig. 2b). No association was found between the levels of Tc17 cells and other clinical characteristics such as clinical stage, infiltration depth, vasoinvasion, histological tumor type, or primary tumor size in UCC patients (*P*>0.05).

### Distribution of Tc17 cells and the microvessel density (MVD) in tissue samples of UCC and CIN patients

**Figure 3:**
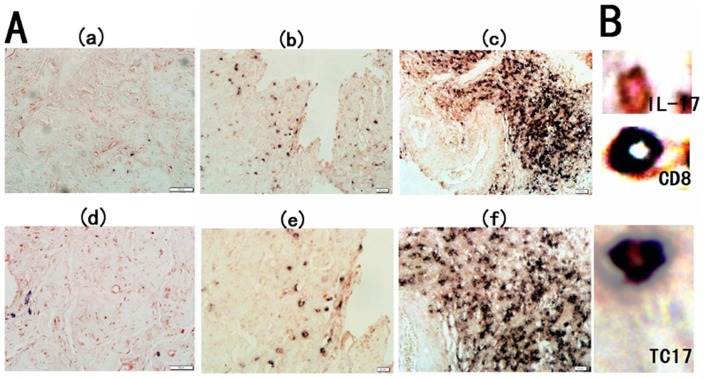
Expression of Tc17 cells in cervical tissues of the three groups. (A) Immunohistochemical double staining for Tc17 cells in the control group (a and d), CIN group (b and e) and UCC group (c and f). Representative sites with low (200×, upper panels) and high (400×, lower panels) magnification were shown. (B) IL-17-producing cells were stained red (in the cytoplasm) and CD8^+^ cells were stained black (in the membrane). The co-expression of CD8 and IL-17 confirmed that a proportion of Tc17 cells.

**Figure 4:**
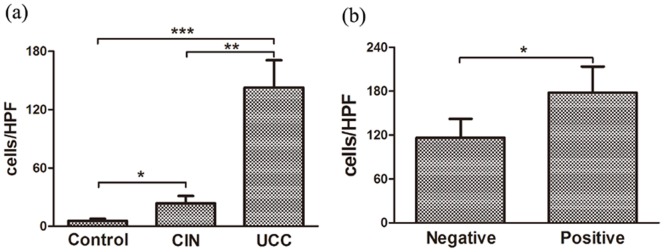
The frequency of Tc17 cells by immunohistochemical staining. (a) Compared to healthy controls (n = 30), significantly increased numbers of Tc17 cells were found in both tissues of UCC patients (*P* = 0.0007, n = 46) and CIN patients (*P* = 0.026, n = 28). Significant difference was also found between CIN and UCC tissues (*P* = 0.0086). (b) In tumor region, those UCC patients with lymph node metastases (n = 30) were detected significantly statistical higher Tc17 cells frequency than those patients without lymph node metastases (*P* = 0.035, n = 16). *P<0.05, **P<0.01, ****P<0.001.

We previously found that IL-17^+^ cells were enriched in human UCC, and most of the circulating IL-17^+^ cells were CD4^+^ cell population (Th17 cells) [8]. However, in tumor tissues, a more abundance of the cells were CD8^+^ T cells (Tc17 cells). To study the distribution of Tc17 cells on a local scale, we examined the prevalence of Tc17 cells in tumor tissues from 46 UCC patients and cervical tissues from 28 CIN patients using immunohistochemical double staining (Fig. 3). As shown in Fig. 4a, the level of Tc17 cells significantly increased in tissues of UCC patients (142.50±28.24 cells/HPF) and CIN patients (23.42±7.65 cells/HPF), when compared to that in tissues of healthy controls (5.52±2.25 cells/HPF; *P*_UCC_ = 0.0007, *P*_CIN_ = 0.026). A significant difference was also found between CIN and UCC patients (*P* = 0.0086). Again, the level of Tc17 cells in tissues of UCC patients was positively correlated with the status of lymph node metastases, since significantly higher level of Tc17 cells was observed in tissues from UCC patients with lymph node metastases (178.20±35.47 cells/HPF) compared to that in UCC patients without lymph node metastases (116.59±25.57 cells/HPF, *P* = 0.035) (Fig. 4b). No correlation was found between the level of Tc17 cells with the other clinical characteristics such as clinical stage, infiltration depth, vasoinvasion, tumor size and histological tumor type of UCC patients (*P*>0.05).

**Figure 5:**
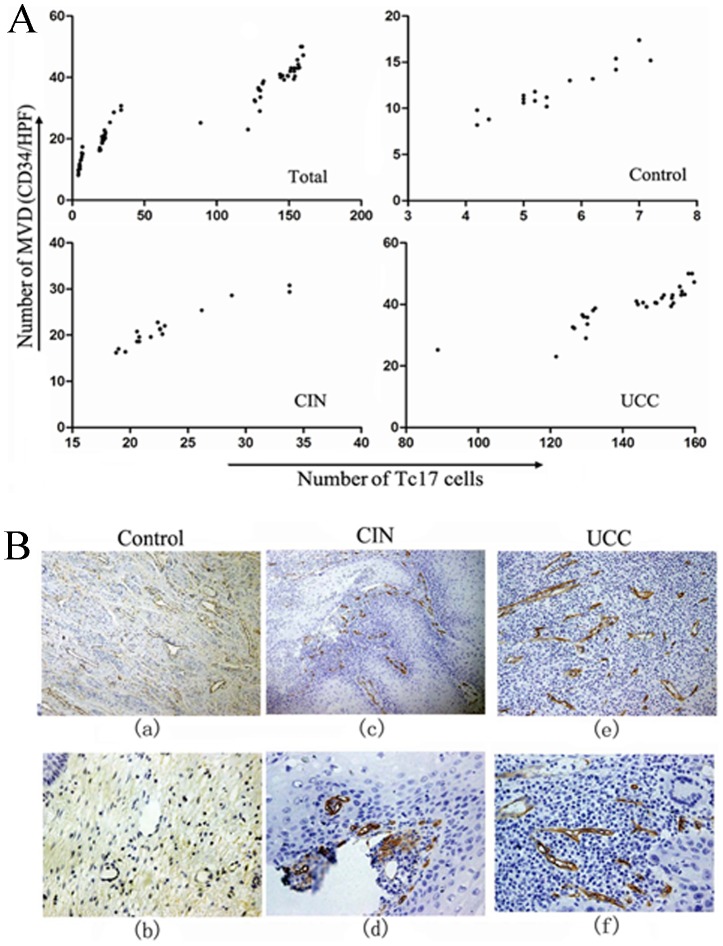
The correlations between Tc17 cells and microvessel density (MVD). (A) Linear regression analysis between the levels of Tc17 cells and MVD (Total: r = 0.987, *P*<0.0001, n = 65; control: r = 0.814, *P*<0.001, n = 18, CIN: r = 0.923, *P*<0.001, n = 17; UCC: r = 0.938, *P*<0.0001, n = 30). MVD from each sample was plotted against Tc17 cells level from the same person. (B) Representative immunohistochemical staining of MVD in cervical tissues of three groups. Representative sites with low (100×, upper panels) and high (400×, lower panels) magnification were shown.

Next, the density of microvessels in cervical tissues was determined by immunohistochemistry staining with antibody against angiogenic marker- CD34. As shown in Fig. 5b, MVD was more frequently observed in UCC (38.37±7.04/HPF, *P*<0.0001) and CIN tissues (23.36±5.34/HPF, *P*<0.0001) than that in the control tissues (11.81±2.47/HPF). Also, significantly higher level of MVD was found in UCC group when compared with CIN group (*P*<0.0001). Since IL-17 is known to be able to promote tumor progression through fostering angiogenesis [21], [22], we analyzed the relationship between the level of Tc17 cells and the amount of microvessels. As shown in Fig. 5a, the levels of Tc17 cells were found to correlate positively with the microvessel density in cervical CIN and UCC tissues (r = 0.923,*P*<0.001 in CIN; and r = 0.938, *P*<0.0001 in UCC, respectively).

### Correlations between the levels of Tc17 cells and Th17 cells as well as Foxp3-expression T cells

**Figure 6:**
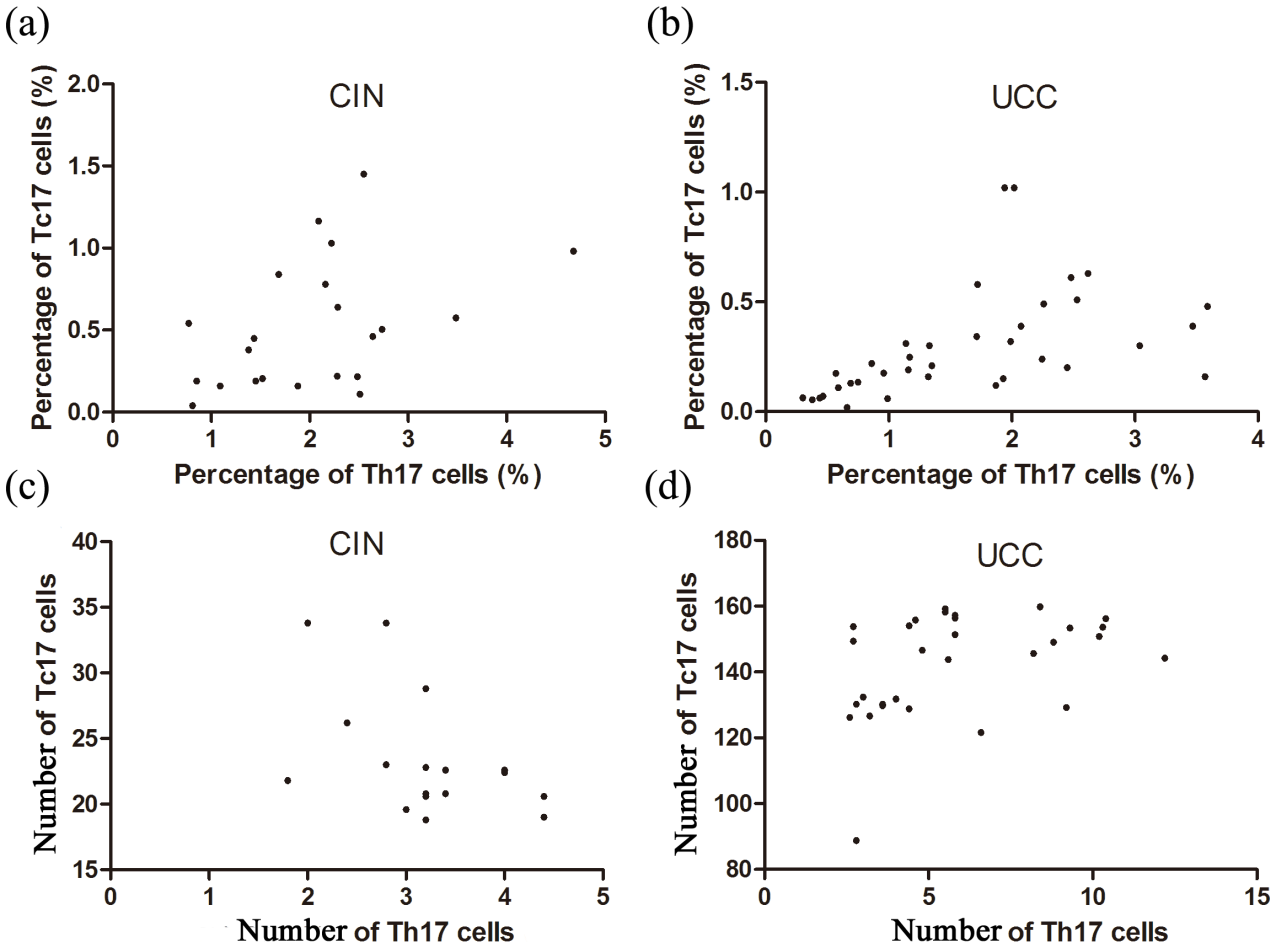
The correlations between Tc17 cells and Th17 cells. (a, b) Linear regression analysis between frequencies of Tc17 cell and Th17 cells in the blood (r_CIN_ = 0.435, *P* = 0.042, n = 22; r_UCC_ = 0.403, *P* = 0.016, n = 36). (c, d) Linear regression analysis between the levels of Tc17 cells and Th17 cells in the cervical tissues (r_CIN_ = 0.441, *P* = 0.039, n = 17; r_UCC_ = 0.693, *P* = 0.026, n = 30).

Since Tc17 cells are similar to Th17 cells in the respect of developmental pathway and possible effects [23], [24], it is reasonable to investigate the relation between them. The primary data and the methods for Th17 cells detection in the same three groups (UCC, CIN and the control groups) were shown in our two previous published papers [8], [25]. The results demonstrated that the levels of Th17 cells and Tc17 cells were positively correlated in the CIN and UCC groups in both peripheral blood (r_CIN_ = 0.435, *P* = 0.042; r_UCC_ = 0.403, *P* = 0.016) (Fig. 6 a and b) and cervical tissues (r_CIN_ = 0.441, *P* = 0.039; r_UCC_ = 0.693, *P* = 0.026) (Fig. 6 c and d).

**Figure 7:**
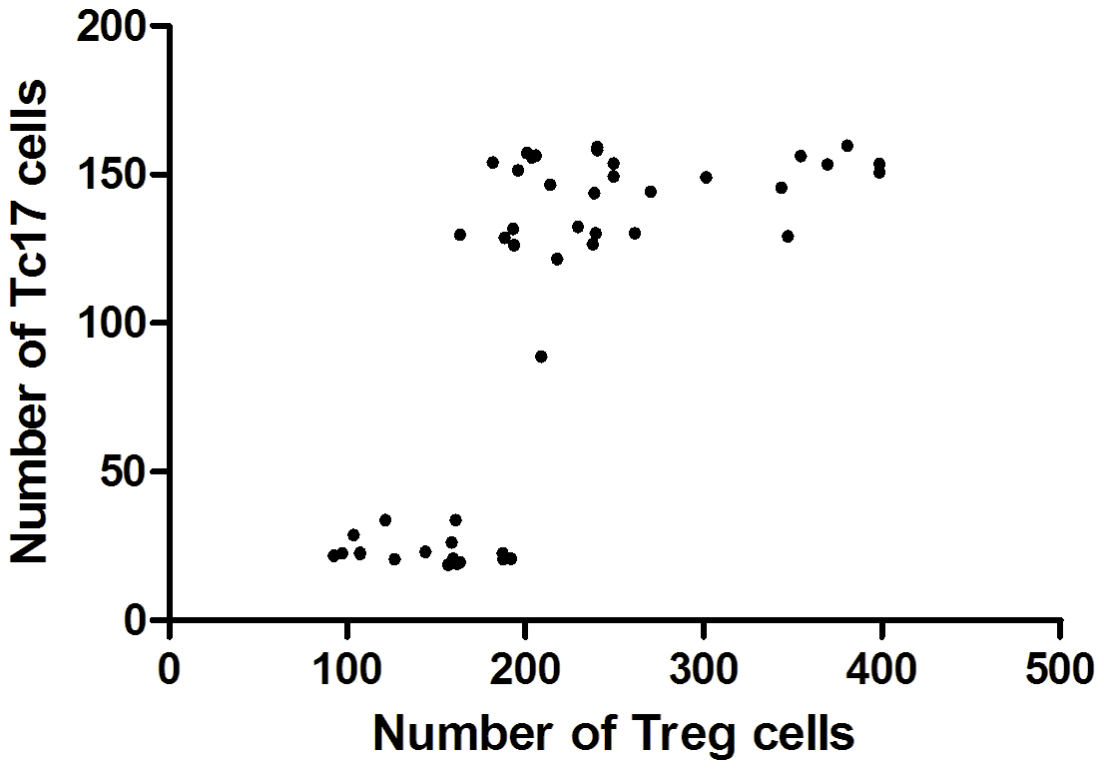
The correlation between Tc17 cells and Foxp3-expressing T cells in the patients of CIN and UCC tissues (r = 0.713, *P*<0.001, n = 47).

Besides, since Foxp3-expressing T cells are well known to be immune-suppressive for the T cell responses [25], [26], the relationship between the levels of Tc17 and Foxp3-expressing T cells in the patient's (CIN and UCC) cervical tissues were also analyzed. The primary data and the methods of Foxp3-expressing T cells detection were in the reference 38. As shown in Fig. 7, the levels of Tc17 cells and Foxp3-expressing T cells in the patient's cervical tissues were positively correlated (r = 0.841, *P*<0.001).

## Discussion

The immune system plays a complex role in tumorigenesis. It was previously believed that CD8^+^ T mainly comprise CD8^+^ cytotoxic T cells, Tc cells, which play crucial role in host immune responses to intracellular pathogens and tumor formation by means of the expression of IFN-γ, granzyme B and perforin [13], [14]. However, a subset of CD8^+^ T cells with immunosuppressive properties have been described [27], [28]. Roberts et al. [29] found that CD8^+^ T cells could also promote the cutaneous carcinogenesis using a two-step chemical initiation/promotion protocol (DMBA and TPA). A DMBA/TPA carcinogenic regimen could up-regulate the expressions of TGF-β and IL-6, resulting in Tc17 cell skewing [30], [31]. Besides, they also found that a subset of tumor-promoting IL-17^+^CD8^+^ cell population might exist in this model.

Tc17 cells are recently defined as an IL-17-producing CD8^+^ T subset that is distinct from Tc1 and Tc2 cells. Although some advances have been made in the understanding of the differentiation and function of Tc17 cells in some types of human diseases and in the homeostatic regulations [9], [10], [32], little is known about the biological function and mechanism underlying the regulation of Tc17 cells in human tumors. Our study demonstrated that the levels of Tc17 cells and Th17 cells prominently increased in both peripheral blood and tumor tissues of UCC patients. Besides, we found that the frequency of Tc17 cells was positively correlated with that of Th17 cells in both peripheral blood and cervical tissues in patients of CIN and UCC. Another study has also shown an abundance of Tc17 cells in tumor-bearing mice, similar to that in inflammatory condition [18]. Also, Kuang et al. [20] found that IL-17^+^ cells (including Th17 cells and Tc17 cells) accumulated in human hepatocellular carcinomas(HCC) and Tc17 cells were enriched in the invading edge of HCCs. Thus, Tc17 cells enriched in UCC patients may play an important role in the development of this type of cancer.

It was reported that Tc17 cells and Th17 cells shared the similar developmental pathways with regard to the differentiation process [10]. For example, TGF-β, IL-6 and IL-21 could stimulate the differentiation of both Tc17 and Th17 cells [10], [19], [24]. Besides, studies have shown that Tc17 cells may display suppressed cytotoxic function similarly to the function of Th17 cells in autoimmune diseases [23], [33], infection [9], [23] and antitumor immunity [15]. Furthermore, IL-17 is a pro-inflammatory cytokine, which is produced by both Th17 cells and Tc17 cells. IL-17 producing T cells are the effectors involved in inducing inflammation and autoimmune tissue injury [34]. Thus, we speculate that the production of IL-17 may mediate the potential roles of Tc17 cells in cancer progression. However, the forced ectopic overexpression of IL-17 in tumor cells demonstrated conflicting effects on tumor growth: tumor inhibiting [35], [36] versus tumor promoting [21], [37]. Notably, Nam et al. [31] reported that IL-17 suppressed apoptosis in several tumor cell lines *in vitro*, and knockdown of the IL-17 receptor in 4T1 mouse mammary cancer cells enhanced apoptosis and decreased tumor growth *in vivo*. Also, we observed the elevated levels of TGF-β, IL-6, IL-17 and IL-21 in cervical cancers (unpublished observations). These observations suggest that UCC may actively secrete large amounts of TGF-βwhich may induce the differentiation of IL-17^+^ T cells, and the latter may in turn promote the development and survival of cancers in an IL-17-dependent manner [31], although further studies are still needed.

Recently, it has been shown that both Th17 and Tc17 cells are present in the tumor microenvironments of several types of human and mouse tumors [19], suggesting that this may be a relatively common phenomenon in tumorigenesis. Besides, we previously reported that Th17 cells might cause malignant progression and poor survival in UCC patients [8]. In this study, we also observed that the positive correlation between Tc17 cells and Th17 cells increased gradually with the progression from CIN to UCC. Furthermore, the prevalence of Tc17 cells was also correlated with lymph node metastases of UCC. Therefore, it is possible that the enrichment of Tc17 cells promotes the progression of UCC in concert with Th17 cells.

Despite the fact that Tc17 cells are effector cells, they could not kill target cells in the way that classical CD8^+^ T cells do [24]. Epidemiological studies have established that inflammation is associated with tumor progression [38]. Inflammation leads to the production of growth factors, angiogenic factors, and matrix-degrading proteases by leukocytes, which play an important role in tumor promotion and progression [4], [38]. Kuang et al. [20] found that Tc17 cells were predominantly enriched in the invading edge of human hepatocellular carcinoma (HCC). These infiltrating Tc17 cells expressed high levels of proinflammatory cytokines, which promote tumor extension via the inflammatory reaction. The neovascularization is another important hallmark of cancer progression. Proinflammatory cytokines such as IL-17 could also promote tumor growth by fostering angiogenesis [21], [22]. In our study, we also found a positive correlation between the frequency of Tc17 cell and microvessel density, supporting the proinflammatory property of IL-17–producing cells. Moreover, we also observed the correlation of Tc17 cells with Foxp3-expressing T cells in cervical cancer tissues,Foxp3-expressing T cells mediate strong immune-suppressive activity on T cell responses [39], [40]. These results indicate that Tc17 cells may promote tumor progression, but the exact function of Tc17 cells in UCC needs further investigation.

In summary, we showed for the first time that the frequency of Tc17 cells increased obviously in CIN and UCC patients, and the increased prevalence of Tc17 cells was associated with lymph node metastases and microvessel density, indicating an important role for Tc17 cells in the initiation and progression of UCC. A better understanding of the underlying mechanism of IL-17 regulating antitumor immune responses may lead to the development of novel therapeutic strategy for UCC patients.
